# Extracellular vesicles from the CNS play pivotal roles in neuroprotection and neurodegeneration: lessons from *in vitro* experiments

**DOI:** 10.20517/evcna.2023.07

**Published:** 2023-03-29

**Authors:** Isaac Colvett, Hannah Saternos, Christina Coughlan, Anne Vielle, Aurélie Ledreux

**Affiliations:** ^1^Department of Neurosurgery, School of Medicine, University of Colorado Anschutz Medical Campus, Aurora, CO 80045, USA.; ^2^Department of Neurology, School of Medicine, University of Colorado Anschutz Medical Campus, Aurora, CO 80045, USA.

**Keywords:** Extracellular vesicles, exosomes, intercellular communication, neurons, glia, neurodegenerative diseases

## Abstract

Intercellular communication between diverse cell types is crucial for the maintenance of the central nervous system, and exosomes have been shown to play an important role in this process. Exosomes are small extracellular vesicles (EVs) that are released by all cell types and carry cargoes that can elicit downstream effects in recipient cells. Exosomal communication in the central nervous system has been implicated in many neurodegenerative diseases, ranging from Alzheimer’s disease to major depressive disorder. Though there remain many unknowns in the field of EV biology, *in vitro* experiments can provide many insights into their potential roles in health and disease. In this review, we discuss the findings of many *in vitro* EV experiments, with a focus on the potential roles in regulating cell viability, inflammation, oxidative stress, and neurite integrity in the central nervous system.

## INTRODUCTION

In the central nervous system (CNS), cell-to-cell communication is crucial to ensure proper development and maintenance of homeostatic conditions^[1,2]^. Extracellular vesicles (EVs) are membrane-bound carriers packed with proteins, metabolites, and nucleic acids (including DNA, mRNA, and microRNAs), mirroring the physiological status of the cell they originate from^[3]^. First observed 40 years ago^[4]^, EVs have been shown to play a prominent role in many important cellular functions^[5]^, giving way to an exponential increase in EV research in the past two decades. Cell-to-cell communication mediated by EVs has been implicated in many diseases ranging from several types of cancers^[6]^ to mental illnesses such as major depressive disorder^[7]^ and even viral infections affecting the brain and spinal cord^[8]^.

For example, there is emerging evidence that EVs play important roles in remodeling the tumor microenvironment, including modulating macrophage activation and facilitating metabolic reprogramming of tumor-associated tissues^[9]^. Further, EVs have been shown to be important modulators and propagators of inflammation and neuroinflammation^[10,11]^. They are at least partially responsible for the spread of toxic misfolded proteins in many neurodegenerative conditions, including Alzheimer’s disease (AD) and Parkinson’s disease (PD)^[12-15]^. AD is the most common neurodegenerative condition, characterized clinically by memory loss, cognitive impairment, and behavioral alterations. The two major neuropathological hallmarks of AD are neurofibrillary tangles (NFTs), composed of toxic misfolded Tau protein, and amyloid beta (Aβ) plaques^[16]^, leading to neuronal loss. PD is a neurodegenerative movement disorder characterized by the presence of α-synuclein, another toxic misfolded protein causing the degeneration of dopaminergic neurons in the substantia nigra in the midbrain^[17]^. Both AD and PD have been described as types of prion diseases, where misfolded proteins spread from one brain region to another, inducing further aggregation^[18]^. There is accumulating evidence that this spread may be mediated or exacerbated by extracellular vesicle-mediated intercellular communication^[2,19-21]^.

Due to their ubiquity in biological processes, EVs are also being extensively studied for their therapeutic potential. Their ability to cross many membranes, including the blood-brain barrier^[22]^, makes them appealing candidates for both non-invasive biomarker assessment and effective drug delivery devices^[23]^. Utilizing proteomics and machine learning, Muraoka *et al.* identified four EV-associated proteins (ANXA5, VGF, GPM6A, and ACTZ) that could distinguish AD patients from control patients with 88% accuracy, illustrating how powerful of a tool EVs can be in the context of biomarkers and therapeutics^[24]^.

However, there is still much that is unknown about EV biology, and studying cell-cell communication through EVs has proven challenging given the variety of mechanisms involved^[25]^. For this reason, *in vitro* studies are crucial to understanding the biological mechanisms involved in EV formation, secretion, and uptake during normal conditions and cell-specific pathological events. Moreover, identifying molecular mechanisms involved in effecting changes in recipient cells could help identify targets for therapeutic drug development. In this review, we aim to provide an overview of current knowledge about the effects of EVs on recipient cells, with a focus on CNS-specific cell types. We first introduce general concepts on EV biogenesis, mechanisms of uptake, and current technology used to study EVs before focusing on *in vitro* studies investigating the effects of EV exposure on cells of the CNS in terms of cell viability, neuroinflammation, oxidative stress, and neurite integrity.

## EXOSOME BIOGENESIS

All cell types, including those of the CNS, are capable of forming and secreting EVs^[26-29]^. Though new categories are being described, EVs can be generally subdivided, based on their size, into exosomes (30-200 nm), microvesicles (MVs, 100-1,000 nm), and apoptotic bodies (> 1,000 nm), each one stemming from different intracellular pathways^[30]^. The defining feature of exosomes is their endosomal origin, differentiating them from apoptotic bodies and MVs (also called ectosomes), which originate from the blebbing of the plasma membrane^[31]^.

Studies have suggested that small ectosomes or microvesicles that bleb directly from the plasma membrane can share many characteristics with exosomes^[32]^. This can make distinguishing true endosomal-origin exosomes from these small ectosomes extremely difficult, especially given that these small ectosomes seem to form at a higher rate than exosomes^[32]^. Since most of the studies compiled for this review likely dealt with a mixed population of EVs, we will use the terms “extracellular vesicles” and “exosomes” interchangeably when discussing the effects of “exosome” treatment on recipient cells, mostly following the language used in the cited literature.

With respect to biogenesis, true exosome formation can occur through an endosomal sorting complex required for transport (ESCRT) dependent or independent pathway^[33]^. In the ESCRT-dependent pathway, large, multi-subunit proteins bind to ubiquitinated proteins while causing inward budding of the endosomal membrane. Each complex in the ESCRT pathway has several ubiquitin-binding domains, which sort ubiquitinated proteins into the intraluminal vesicles (ILVs) and curate the cargo of exosomes^[30]^. Since ESCRT-dependent exosome formation is the most common form of exosome biogenesis^[34]^, ESCRT-associated proteins such as ALIX and TSG101 are commonly used for exosome characterization^[25]^.

However, ILVs are still formed when cells are depleted of ESCRT machinery^[34]^, indicating that ESCRT-independent pathways still play major roles in exosome formation. For example, ceramide and its metabolite sphingosine-1-phosphate have been shown to be critical in sorting cargo into ILVs, independent of ESCRT machinery^[33]^. In this model, membrane proteins are retained into cholesterol rafts, while the increased accumulation of ceramide due to sphingomyelinase causes the inward budding of the endosomal membrane, forming ILVs without the use of ESCRT machinery^[35]^. Interestingly, all brain cell types have been shown to be at least partially dependent on ESCRT-independent exosomal formation^[13,36,37]^.

In either the ESCRT-dependent or independent pathways, how the multivesicular body (MVB) is transported to the plasma membrane to secrete these exosomes is not well understood, though many Rab GTPases may play crucial roles. Ostrowski *et al.* conducted an RNA interference screen in HeLa cells and found that silencing Rab27a and Rab27b decreased exosome secretion^[38]^. Additionally, knocking down a stabilizer of Rab27a has been shown to cause a decrease in exosome secretion along with an increase in MVB size in neuronal cell lines, making these pathways relevant for the study of brain exosomes^[39]^.

Due to their high levels of compartmentalization, polarized morphology, and excitability, exosome biogenesis and release in neurons are more nuanced than in other cells. Indeed, exosomes released from the axonal region of the neuron may carry different cargoes than exosomes released from the somatodendritic compartment^[40]^. Neurons are also highly excitable cells, and exosome release is regulated by synaptic activity^[41]^, adding another layer of complexity to exosome biogenesis and secretion in neurons.

## MECHANISMS OF EXOSOME UPTAKE

**Figure 1:**
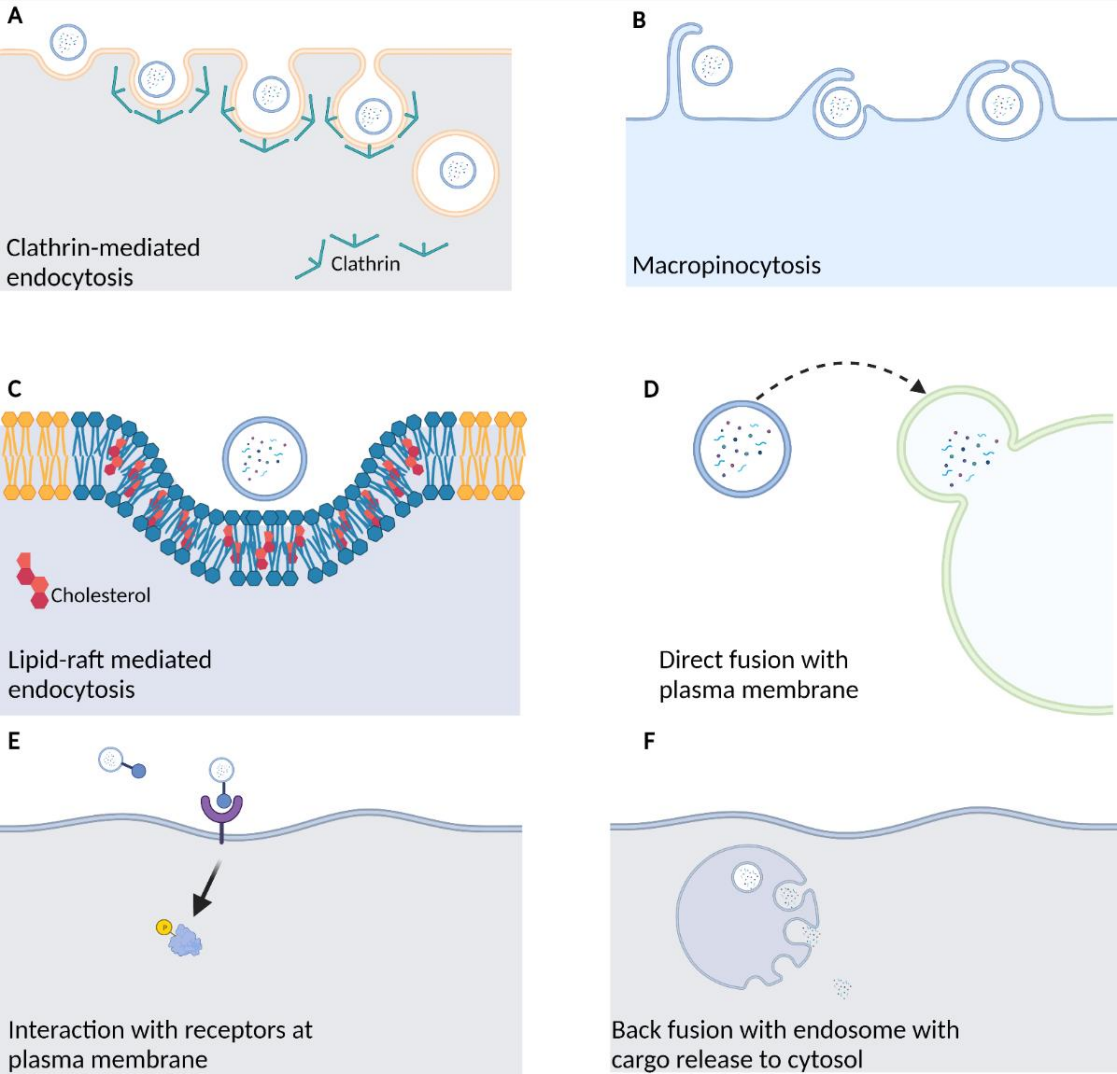
Extracellular vesicles can be internalized into recipient cells by different mechanisms. Image created with BioRender.

The two most common forms of exosome uptake into the recipient cell are clathrin-mediated endocytosis^[42,43] ^[Figure 1A] and macropinocytosis^[44] ^[Figure 1B]. In clathrin-mediated endocytosis, the exosomes bind to the plasma membrane and are then internalized into endosomes. Once inside the endosomal pathway, they can either be degraded in the lysosome^[45]^ or undergo lysosomal escape and release their cargo into the cytoplasm^[46]^. Exosomes can also undergo back-fusion with the endosomal membrane [Figure 1F], providing another pathway for the delivery of functional cargo into the cytoplasm^[42]^. Macropinocytosis is a mechanism where the plasma membrane ruffles out in an actin-dependent manner and internalizes extracellular components. This has been shown to be a major form of exosome internalization in microglia, as these cells are known to perform constitutive macropinocytosis^[44]^.

Other exosome internalization mechanisms appear to involve lipid raft-associated endocytosis [Figure 1C], as disruption of these structures by altering cholesterol dynamics can potently inhibit exosome uptake^[47]^. Mechanisms such as these may account for observations of exosome uptake independent of macropinocytosis and clathrin-mediated endocytosis^[48]^. Other forms of uptake include direct fusion with the plasma membrane [Figure 1D] and phagocytosis^[14,48]^. Additionally, exosomes can cause changes in recipient cells without internalization, likely through contact between integral proteins on the exosome membrane and proteins on the plasma membrane^[49] ^[Figure 1E]. Since the cellular changes caused by exosome uptake can vary depending on which uptake mechanism is at play^[14,15]^, understanding more about exosome uptake is crucial for the development of therapeutic strategies targeting this process.

Although all cell types can form and secrete exosomes, their uptake appears to be somewhat selective. Whether exosomes are taken up by the recipient cell can depend on a variety of factors, including donor and recipient cell types. Recent studies suggest that astrocytes cannot take up exosomes from oligodendroglia or microglia under healthy conditions^[50]^. Furthermore, while neuroblastoma cell lines produce exosomes that can be taken up by microglia, other neurons, and astrocytes, some studies report that primary neurons produce exosomes that are only taken up by other neurons^[51]^, with some debate as to whether astrocytes can take them up^[51,52]^. Microglia, though, appear to internalize exosomes from a wide range of cell types efficiently, and they have been shown to take up exosomes from oligodendrocyte precursor cells^[44]^, neuroblastoma cells^[53]^, primary neurons^[10]^, astrocytoma cells^[54]^, primary astrocytes^[55]^, immortalized microglia cells^[56]^, and other primary microglia^[57]^. This is fitting, as microglia are commonly referred to as the sentinels of the brain^[58]^. Overall, the mechanisms underlying recipient cell-targeting by EVs as well as cell-dependent internalization of EVs remain somewhat elusive. It appears that the surface composition of the EVs can greatly influence the specific targeting of recipient cells. EV surface molecules such as lipids, tetraspanins, integrins, and glycoproteins have been proposed to play a role in EV tropism^[30]^. Studies also suggest that integrins selection and display on EV surface play a major role in EV targeting specific cell types^[59]^.

## TOOLS USED IN EXOSOME RESEARCH

Despite the increase in exosome research over the last decade, there are still many remaining challenges in the isolation, characterization, and tracking of exosomes. Various enrichment methods yielding different “purities” of exosomes have been described^[60]^, and no one-size-fits-all method of enrichment has emerged thus far^[25]^. Though methods of molecular characterization of exosomes such as Western blotting, ELISA, and mass spectrometry have been used extensively, these are susceptible to contamination from other sources, and systematically identifying subpopulations based on the presence or absence of proteins remains elusive^[61,62]^. In terms of exosome tracking, while lipid dyes such as PKH26 have been widely used, they can form aggregates in fixed cells in the size range of exosomes, giving false positives in microscopy studies. Further, evidence suggests that cargo labeling may affect the formation and properties of the exosomes themselves, depending on the labeling method used^[63]^.

Methods for enrichment or separation of exosomes normally rely on their size, density, or surface proteins. Therefore, they consist of size-exclusion chromatography (along with additional concentration steps), differential ultracentrifugation, and immunocapture, respectively, each with different benefits and drawbacks^[64]^. Innovative methods have been developed that could potentially bring better consistency and reproducibility to the exosome field. For instance, Yang *et al.* utilized an artificial antibody known as a “molecularly imprinted polymer” to magnetically isolate EVs, which resulted in a 3-fold enrichment of plasma exosomes compared to ultracentrifugation^[65]^. This method also has the potential to be customized for different capture probes with high specificity and may present a novel and precise isolation technique^[66]^.

Tracking exosome secretion, uptake, and fate in recipient cells is also becoming more feasible with the development of innovative tagging strategies. Sung *et al.* recently reported a stable and more brightly fluorescent version of the molecule known as pHluorin for studying exosome formation and secretion^[67]^. Under acidic conditions, such as in the lysosome or MVB, pHluorin is non-fluorescent, only emitting fluorescence at neutral pH conditions, such as in the extracellular space^[67]^. Tagging the CD63 tetraspanin, a common marker for exosomes mainly found in EVs secreted from the endosomal pathway^[32]^, with this new version of pHluorin, enables the visualization of exosome secretion^[67]^. This is important because there has been evidence showing that small ectosomes budding directly from the plasma membrane can share almost all of the same characteristics as true endosomal-origin exosomes while potentially forming in much higher numbers than true exosomes^[32,62]^. Tools like pHluorin that label proteins specifically based on their subcellular location can potentially help differentiate between these subpopulations of EVs.

Using the extremely bright and small bioluminescent protein nanoluciferase, O’Brien *et al.* were able to track the fate of internalized exosomes with high resolution, allowing them to analyze how internalized exosomes can transfer bioactive cargo to recipient cells. They found that the transfer of bioactive cargo is rare and inefficient, and their methods in tracking uptake and fate generated precise data that can be used in further studies^[68]^.

## THE EFFECTS OF EXOSOMES ON CELL VIABILITY

**Figure 2:**
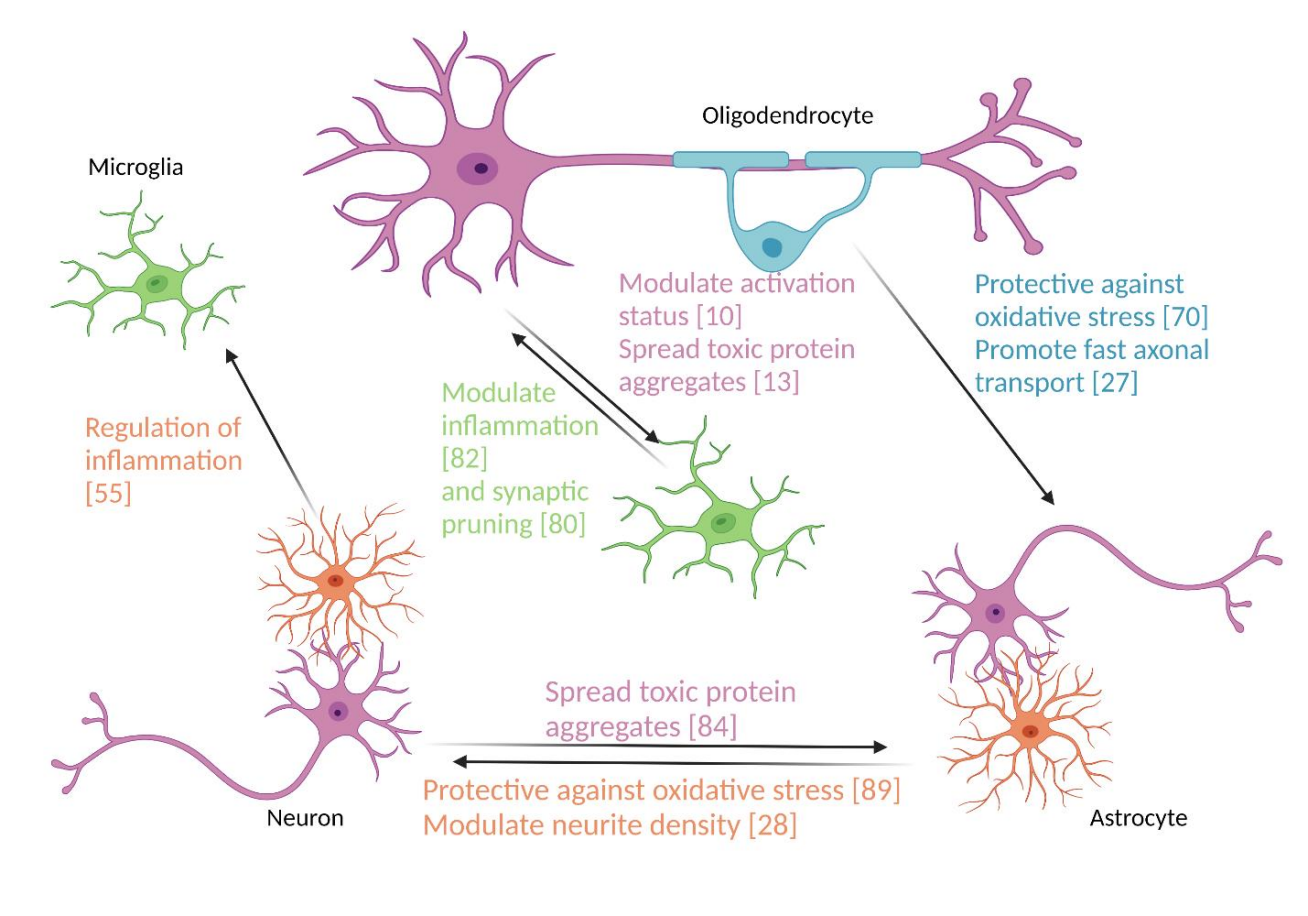
Extracellular vesicles contribute to intercellular communication in the central nervous system. Image created with BioRender.

Typical measurements of cell viability include metabolic assays that use mitochondrial activity and membrane integrity as indicators of the number of living cells in a culture, as well as exclusion dye experiments that mark intracellular structures that are not enclosed in a membrane in non-viable cells as with propidium iodide which stains the DNA of non-viable cells^[69]^. Cell viability is one of the broadest measurements of cell health, as it simply measures the number of living cells in a population without identifying the potential mechanisms of any reduced viability. Despite these limitations, cell viability is an important starting place for studying how certain populations of exosomes elicit effects in recipient cells [Figure 2]. Here, we will focus mostly on how the cargo of glial cell exosomes affects neuronal viability [Table 1].

**Table 1:** Effects of EVs on cell viability

**Donor cells**	**Recipient cells**	**Result**	**Reference**
Mouse primary oligodendrocytes	Mouse primary neurons 12-14 hrs before oxidative stress	Increased viability	[70]
SH-SY5Y cells with the Swe mutation of APP	CHME3 microglia	Increase in necrotic bodies initially, with a delayed increase in apoptosis	[53]
Total plasma EVs from AD patients	Primary rat neurons	No difference in viability	[28]
Astrocyte-derived plasma EVs from AD patients	Primary rat neurons	Decreased cell viability not due to apoptosis	
	hiPSC neurons		
Astrocyte-derived plasma EVs from FTLD patients	Primary rat neurons	No difference in viability	
	hiPSC neurons		
Astrocyte-derived plasma EVs from mTBI patients	PC12	Increased cytotoxicity	[72]
	SH-SY5Y		
EVs from healthy control brain tissue	hiPSC neurons	Increased cytotoxicity	[71]

AD: Alzheimer’s disease; APP: amyloid precursor protein; EVs: extracellular vesicles; FTLD: frontotemporal dementia; mTBI: mild traumatic brain injury.

In general, it seems as though exosomes from healthy donor glia are not inherently harmful to the survival of recipient neurons *in vitro *and may even elicit beneficial effects. When primary neurons were treated with exosomes from primary oligodendrocytes 12 to 14 h before undergoing an oxidative stress challenge, they exhibited substantially higher metabolic activity, indicating higher viability, when compared to treatment with exosomes from HEK293 cells, which suggests a specific function of oligodendrocyte-derived exosomes for neuronal survival of oxidative stress^[70]^. It is worth noting that different cell types may be more affected by exosomes than others. Indeed, exosomes enriched from the brain tissues of healthy humans caused increased cytotoxicity in mature neurons derived from hiPSCs but not in differentiated SH-SY5Y neuroblastoma cells^[71]^. So, while exosomes from healthy donor cells may have no effect or may even be beneficial to some recipient cells, they may also be detrimental to others, highlighting the importance of utilizing strategic controls in viability experiments with exosomes and demonstrating the complex interactions of exosomes with recipient cells.

When exosomes come from diseased donor cells, the response of recipient cells can be even more varied. In particular, astrocyte- and neuron-derived exosomes isolated from the plasma of people with mild traumatic brain injury (mTBI) caused increased cytotoxicity in recipient PC12 and SH-SY5Y neuroblastoma cells, suggesting that alterations in both neurons and glia in the brain during TBI can be propagated via exosomes^[72]^. Similarly, astrocyte-derived exosomes (ADEVs) from patients with AD caused decreased cell viability in recipient primary rat neurons as well as hiPSC neurons, while total serum EVs from these patients had no effect on viability, even despite the species difference. This finding was linked to high levels of complement proteins, specifically the membrane attack complex proteins, in the ADEVs and neuron-derived EVs (NDEVs), suggesting that these cargoes were crucial for the detrimental effects observed. Interestingly, this study also showed that ADEVs from patients with fronto-temporal lobular degeneration caused no decrease in cell viability in these same cultures, further pinpointing these toxic effects on AD pathology. This suggests that exosome effects can also be highly specific to the disease and donor/recipient cell types. Additionally, while there was a significant increase in necrosis in the recipient cells, there was no evidence of increased apoptosis^[28]^. This is consistent with a study by Fernandes *et al.* showing that exosomes from SH-SY5Y cells expressing the Swedish mutation (Swe) of APP (causing the cells to accumulate more Aβ plaques) increased necrotic bodies in their recipient CHME3 microglia when compared to exosomes from control SH-SY5Y cells during the exosome incubation period^[53]^. However, Fernandes *et al.* added a condition where they isolated exosomes from the cells and continued to observe them *in vitro* for another 24 h and noted that even after this additional exosome-free incubation time, there was an increase in late apoptotic markers^[53]^, suggesting that exosomes from patients with AD may also eventually cause an increase in apoptotic responses while initially only increasing necrosis in recipient glial cells.

Moving forward, it will be important to couple these studies of how EVs can cause these changes in cell viability with the mechanisms involved in their uptake. Some studies have shown that, in the context of AD, the digestion of proteins on the surface of EVs can abrogate any changes in cytotoxicity caused by these EVs^[28]^, while others have demonstrated a toxic effect of mainly luminal proteins^[71]^. While both factors on the exosome surface and in the exosome lumen can be considered “exosomal cargo”, it remains unknown to what extent these exosomes are decreasing viability through the delivery of luminal cargoes or through binding to receptors on the plasma membrane. Therefore, while exosomes containing specific pathologies can affect the viability of recipient cells, whether this occurs and by what mechanism it occurs depends on the cell types and disease states of both donor and recipient cells. Some of the specific pathways and mechanisms that may underlie these effects are reviewed in the next sections. 

## INFLAMMATION AND EXOSOMES

The inflammatory effects exosomes have on their recipient cells depend on the cell type, inflammatory status, and disease state of both the donor and recipient cells. Due to this high amount of contextual variability, it remains difficult to draw significant conclusions. Nonetheless, there are some noteworthy trends (summarized in Table 2 and Figure 2).

**Table 2:** Effects of EVs on Inflammation

**Donor cells**	**Recipient cells**	**Result**	**Reference**
Primary rat neurons	Primary rat microglia	Reduction in M1 and M2 activation	[10]
		Protective against future LPS-stimulated activation	
PC12 neurons	MG6 microglia	Upregulation of complement factor 3	[78]
SH-SY5Y neurons with the Swe mutation of APP	CHME3 microglia	Increased secretion of pro-inflammatory cytokines	[53]
Serum-derived exosomes from patients with major depressive disorder	BV2 microglia	Increased activation	[7]
Microglia transfected with miR-124 mimic	Primary mouse neurons	Inhibition of inflammatory response	[80]
Primary astrocytes treated with brain extracts from a mouse TBI model	Primary mouse microglia	Promotion of M2 activation state	[55]
Primary neurons	Primary mouse microglia	Transfer of miR-124 promoting M2 activation	[81]
	Primary mouse astrocytes		
BV2 microglia in M2 activation	Primary mouse neurons	Increase of miR-124 expression	[82]
SH-SY5Y neurons treated with methamphetamine	U87MG astrocytes	Increased inflammatory response that is abrogated by knocking out α-synuclein	[84]
Brain extracts from patients with rTBI	BV2 microglia	Increased expression of miR-124, promoting M2 activation	[80]

APP: Amyloid precursor protein; rTBI: (repetitive) traumatic brain injury; LPS: lipopolysaccharide.

Microglia are important mediators of the immune response in the CNS. Microglia are constantly surveying their surroundings^[73]^ and are vital to a diverse array of brain functions such as memory formation and synaptic pruning^[74]^. These roles require a constant and delicate balance between pro-inflammatory and anti-inflammatory factors^[75]^. This dynamic nature of microglia also extends to “activated” microglia, which respond to injury and disease through a wide spectrum of different activation states^[75]^. Broadly, though, there are two different states of microglial activation: the M1 and M2 states^[76]^. While this paradigm has been challenged in recent years^[77]^, many studies we looked at used this terminology so that we will use the language from the literature cited as much as possible. In the M1 state, the microglia generally release pro-inflammatory cytokines and produce high amounts of reactive oxygen species, whereas, in the M2 state, the microglia generally release anti-inflammatory cytokines and engage in tissue and extracellular matrix repair^[76]^. Though the inflammation response in the brain is highly complex and broadly unknown, there is substantial evidence that exosomes are important in modulating microglial activity and activation.

In healthy states, neuronal exosomes may be key players in keeping microglia in a quiescent, non-inflammatory state, as exosomes from primary neurons were observed to reduce both M1 and M2 activation of recipient primary microglia and were protective against future lipopolysaccharide (LPS)-stimulated activation of the microglia^[10]^. In addition, PC12 neuronal cell exosomes caused an upregulation of complement factor 3 in MG6 microglia cells, which was demonstrated to be an important step in the ability of microglia to perform synaptic pruning, potentially indicating a beneficial level of inflammation^[78]^. These findings point to the important role of neuronal exosomes in balancing the inflammatory and resting states of microglia.

Exosomes enriched from pathology-containing tissues or cells elicit different effects on recipient cells, depending on the disease. In the case of AD, SH-SY5Y neurons engineered to express the Swe mutation of APP produced exosomes that increased the secretion of pro-inflammatory cytokines in CHME3 microglia^[53]^. This is consistent with other studies that have shown a correlation between Aβ levels and neuroinflammation^[79]^. Serum-derived exosomes from patients with major depressive disorder were also able to cause activation in recipient BV2 microglia cells^[7]^. Moreover, exosomes from a well-established *in vitro* model of depression (PC12 neurons treated with 200 mM of corticosterone), when stereotaxically injected into the hippocampus of male C57BL/6J mice, were shown to induce depression-like behaviors, as demonstrated by avoidance of the central area in an open field test. This effect was attributed to the microRNA miR-9-5p promoting microglial M1 polarization through down-regulating the protein suppressor of cytokine signaling 2 (SOCS2)^[7]^.

In the case of TBI, BV2 microglia cells exposed to brain extracts from a repetitive traumatic brain injury (rTBI) mouse model exhibited a higher expression of microRNA miR-124-3p, which was found to promote the M2 anti-inflammatory state^[80]^. Jiang *et al.* found that neuronal exosomes could carry miR-124-3p to recipient astrocyte and microglia cells after spinal cord injury, suppressing their activated state^[81]^. However, other studies also demonstrated that astrocytes could promote the M2 activation state of microglia in TBI through the microRNA miR-873-5p, demonstrating that multiple cargoes could potentially be causing this effect after neurotrauma^[55]^. More research involving neuron-derived exosomes from *in vivo* models of TBI and CNS injury would be useful in identifying mechanisms affecting the activation response of microglia. 

Exosomal miR-124 seems to also have a beneficial effect when communicated from microglia to neurons since exosomes from BV2 microglia in the M2 anti-inflammatory phenotype could increase the expression of miR-124 in recipient primary neurons, which was shown to be important in increasing neuronal survival under oxygen/glucose deprivation^[82]^. Huang *et al.* found that microglia transfected with a miR-124-3p mimic could inhibit the inflammatory response of primary cortical neurons by inhibiting mTOR^[80]^. This suggests a potential positive feedback loop, where cells packing miR-124 into exosomes can cause further expression of miR-124 in other surrounding cells.

Though astrocytes are also key regulators of immunity in the CNS, many studies report rare or no exosome uptake at all in astrocytes^[44,50,51,83]^. Nevertheless, it seems that under pathological conditions, they may be able to take up exosomes from some neurons. Specifically, primary neurons treated with methamphetamine produced exosomes that elicited an inflammatory response in recipient primary astrocytes, demonstrated by an increase in the production of pro-inflammatory cytokines like TNF-α, IL1β, and IL6 after exosome treatment^[84]^. However, this observed uptake may be due to the use of a neuroblastoma cell line, as another group found that astrocytes take up exosomes from N2A cells but not from primary neuronal cells^[51]^. Despite being key players in the regulation of neuroinflammation, much is unknown about whether and to what extent astrocytes modulate the inflammatory response of neurons through exosomes.

Although this review is focused on *in vitro* experiments, it is important to note that many similar trends are observed *in vivo *as well. Li *et al.* found that exosomes from mice stimulated with LPS, when administered intravenously to other mice, could induce microglial and astrocytic activation^[85]^. Our group showed that NDEV enriched from the plasma of individuals with Down syndrome elicited elevated neuroinflammation when injected into the hippocampus of wild-type mice^[20]^. Furthermore, exosomes from M2 microglia containing miR-124 were able to protect against neural deficits and neuronal apoptosis in a mouse model of stroke^[82]^. In a mouse model of spinal cord injury, NDEVs containing miR-124 were able to suppress excessive activation of microglia and astrocytes *in vivo*^[81]^. The authors linked these neuroprotective, anti-inflammatory effects of miR-124 to its ability to suppress myosin heavy chain 9, a protein known to increase inflammatory cytokine production^[81]^.

Overall, these findings point to some trends in the complex interplay between exosomal communication and the regulation of neuroinflammation (summarized in Table 1 and Figure 1). Owing to the variability across experimental systems, it remains difficult to draw any significant conclusions, and more studies are needed to explore how exosomes communicate inflammatory responses across cell types as well as what mediates these effects. Specifically, teasing out what inflammatory responses are caused by the luminal cargo of exosomes derived from the cytoplasm of their donor cell and which responses are caused by potential surface cytokines the exosomes picked up extracellularly will be crucial.

## OXIDATIVE STRESS AND EXOSOMES

The process of oxidative phosphorylation is vital for generating energy in mammalian tissues; however, as a by-product of this biochemical pathway, many reactive oxygen species (ROS) and reactive nitrogen species (RNS) are produced^[86]^. In brain tissue under normal conditions, these ROS and RNS molecules are important in processes such as neuronal development, long-term potentiation, and overall health, as the tissue’s antioxidant system prevents these reactive species from damaging the cell. However, under diseased conditions, this balance is disrupted, and these reactive species become toxic^[86]^. Because the brain is rich in peroxide-susceptible lipids and has a high energy demand, the CNS is particularly vulnerable to oxidative stress. Accordingly, oxidative stress is a feature of many neurodegenerative conditions^[87]^. Oxidative stress alters both the number of exosomes released as well as the cargo of these exosomes, and exosomal communication can confer both beneficial and detrimental effects on recipient cells^[88]^, as summarized in Table 3.

**Table 3:** Effects of EVs on oxidative stress

**Donor cells**	**Recipient cells**	**Results**	**Reference**
1321N1 human astrocytes	SH-SY5Y neurons undergoing oxidative stress	Increased viability	[90]
Primary astrocytes from mouse midbrain, activated with CCL3	SH-SY5Y neurons undergoing oxidative stress	Reduction in apoptosis	[92]
	SH-SY5Y neurons injured with MPP^+^	Preservation of mitochondrial complex I	
Glioblastoma cells	Primary rat neurons	Increase in oxidant status and decrease in antioxidant status	[94]
BV2 microglia exposed to NfL	Primary rat neurons	Increase in oxidative stress	[95]
		Decrease in viability	
Serum exosomes from a mouse model of Parkinson’s disease	Primary mouse neurons	Downregulation of OXR1 through the miRNA cargo miR-137	[93]

CCL3: Chemokine (C-C motif) ligand 3; MPP^+^: 1-methyl-4-phenylpyridinium; NfL: neurofilament-light; OXR1: oxidation resistance 1.

Astrocyte-derived exosomes are particularly crucial for protecting neurons against oxidative stress as they have been shown to carry prion protein, synapsin I, heat shock protein (HSP70), superoxide dismutase (SOD1), and catalase, all of which are important components of the antioxidant system in the neuron^[89]^. Furthermore, Pascua-Maestro *et al.* found that apolipoprotein D is protective against oxidative stress and is exclusively transported to neurons through exosomes^[90]^. This was demonstrated by the increased viability of SH-SY5Y neurons undergoing oxidative stress after being treated with the EV fractions of 1321N1 astrocytes^[90]^.

This function of ADEVs may be vital to understanding certain neurodegenerative conditions. For example, exosomes from primary rat astrocytes decreased cognitive impairment, neuronal loss, and ROS levels when stereotaxically injected into a rat TBI model while increasing antioxidant molecules such as SOD1 and reduced glutathione^[91]^. Additionally, primary astrocytes isolated from the mouse ventral midbrain and striatum, when activated with the dopaminergic neuron protector molecule chemokine ligand 3 (CCL3), produced exosomes that reduced apoptosis of SH-SY5Y neurons undergoing oxidative stress^[92]^. This same research found that these exosomes could preserve the activity of mitochondrial complex I in SH-SY5Y cells injured with MPP^+^ (1-methyl-4-phenylpyridinium), a common model for studying PD, illustrating the potentially beneficial role of ADEVs in mitigating neurodegenerative diseases^[92]^.

Exosomes can also propagate the detrimental effects of oxidative stress into other cells. For instance, Jiang *et al.* found that high levels of miR-137 in exosomes derived from the serum of a mouse PD model can downregulate oxidation resistance 1 (OXR1) protein in primary mouse neurons and cause cell death^[93]^. Additionally, glioblastoma cells can release exosomes that increase the total oxidant status while decreasing the total antioxidant status of recipient neurons, causing oxidative stress and increasing cell death^[94]^. This spreading of oxidative stress can also have a self-reinforcing feedback loop, as damaged neurons releasing the neuronal-damage marker neurofilament light chain (NfL) can cause microglia to release more ferritin heavy chain in their exosomes, which in turn increases oxidative stress and decreases cell viability of recipient neurons even more^[95]^.

These studies illustrate the crucial role of exosome communication in both the mitigation and propagation of oxidative stress in the brain. However, there are still unanswered questions. For example, some groups have found that miR-137 can be protective against oxidative stress in astrocytes^[96]^, directly contradicting the findings of the Jiang study, and others have found it to be key in protecting microglia against LPS inflammatory stimulation^[97]^. It is unclear why this particular miR can seemingly have both beneficial and detrimental effects, and studying what determines these opposite effects is crucial to deepen our understanding of exosome biology. Finally, while microglia generate high levels of ROS and contribute to oxidative stress in the brain^[98]^, there has been comparatively little research done on how exosomes modulate microglial oxidative stress. A better understanding of how exosomes and their cargos contribute towards and fight against oxidative stress is important, considering how prevalent oxidative stress is in many neurodegenerative conditions.

## NEURITE INTEGRITY

The term “neurites” refers to both axons and dendrites, and these structures are crucial to maintaining homeostasis both for individual neurons as well as for the CNS as a whole. Their formation is an actin-dependent process whereby a growth cone extends from the cell body and probes the environment around the cell for permissive elements, leading the axon to a destination where it forms a synapse^[99]^. These synapses then undergo selective elimination to refine neuronal connectivity in a process known as “synaptic pruning”, which occurs both during development and throughout life^[78]^. This is important for the appropriate wiring of neural circuits as well as for behavior, learning, and memory^[78]^. Once synapses are formed, the neuron can use the axon to transport proteins from the cell body out to the synapse (anterograde transport) or from the synapse back to the cell body (retrograde transport), both of which are crucial for neuronal survival^[100]^. Since axons can extend anywhere from a few millimeters to up to a meter away from the cell body, maintaining these structures is an energy- and resource-intensive process^[100]^. Failing to maintain axonal integrity is a feature in many neurodegenerative diseases such as AD, PD, and amyotrophic lateral sclerosis^[101,102]^. There is emerging evidence that exosomal communication in the brain plays an important role in maintaining neurite integrity [Table 4].

**Table 4:** Exosome effects on neurite health

**Donor cells**	**Recipient cells**	**Results**	**Reference**
BV2 microglia transfected with miR-124 mimic	Primary mouse neurons	Increase in neurite outgrowth	[80]
PC12 neurons	MG6 microglia	Increased synaptic pruning activity	[78]
Primary mouse oligodendrocytes	Primary mouse neurons	Promotion of fast axonal transport	[27]
Co-culture of primary mouse neurons, astrocytes, and oligodendrocytes exposed to Aβ fibrils	Primary mouse neurons	Severe synaptic and dendritic loss	[14]
Primary astrocytes stimulated with IL1β	Primary mouse neurons	Reduction in neurite length in a dose-dependent manner	[29]
		No change in total neurites	
Astrocyte-derived plasma exosomes from patients with AD	Primary rat neurons	Decrease in neurite density	[28]
Neuron-derived plasma exosomes from patients with AD		No effect on neurite growth	
Neuron-derived plasma exosomes from TBI patients	PC12 neurons	Decrease in neurite outgrowth	[72]
		Increase in neurite blebbing	

Aβ: Amyloid beta; IL1β: interleukin-1 beta; AD: Alzheimer’s disease; TBI; traumatic brain injury.

Beneficial effects of brain exosomes on neurite integrity include facilitating neurite outgrowth, synaptic pruning, and axonal transport. Exosomes from BV2 microglia cells expressing a mimic of miR-124-3p were able to increase neurite outgrowth of primary mouse cortical neurons^[80]^, suggesting a beneficial effect of glia-derived exosomes on neurite outgrowth. MG6 microglia cells treated with rat PC12 neuroblastoma exosomes also performed more synaptic pruning when later co-cultured with the PC12 neurons^[78]^, indicating that neuron-to-glia exosomal communication can also be an important mediator of synaptic pruning. Finally, exosomes from primary oligodendrocytes could promote fast axonal transport, reduce pausing time in retrograde and anterograde axonal transport, and even rescue axonal transport in neurons under starving conditions^[27]^. Together, these findings suggest that exosomal communication between neurons and glial cells is important in forming healthy neurites and maintaining axonal homeostasis.

In contrast, exosomes can have detrimental effects on neurite integrity in diseased states. ADEVs from patients with AD reduced neurite density in primary rat cortical neurons^[28]^. This was specific to astrocytes, as NDEVs from the same patients did not elicit effects on neurite outgrowth in this *in vitro* model^[28]^, suggesting that differences in the cargo of ADEVs *vs*. NDEVs result in different outcomes in recipient cells. A co-culture of neurons, astrocytes, and oligodendrocytes exposed to Aβ fibrils for 24 h produced exosomes that caused a severe synaptic and dendritic loss in primary mouse neurons^[14]^, suggesting that the deleterious effects of Aβ fibrils can be transferred through exosome cargo. Inflammation likely plays an important role in these effects, as You *et al.* found that astrocytes stimulated with IL1β caused a reduction in neurite length of primary murine neurons in a dose-dependent manner^[29]^. Interestingly, the authors did not find that the exosomes decreased the total number of neurites *in vitro*^[29]^, which suggests that these exosomes could be specifically targeting pathways involved in neurite outgrowth. These data show compelling evidence for the role of ADEVs in synaptic and dendritic dysfunction in AD.

Though glial exosomes, particularly from astrocytes, are clearly important modulators in neurite integrity and function, NDEVs from patients with TBI were shown to cause a decrease in neurite outgrowth and an increase in neurite blebbing in a neuroblastoma cell line^[103]^, highlighting the potential role of NDEVs in neurite health. 

Replicating these findings *in vivo*, Asai *et al.* found that depleting mouse brains of microglia resulted in a suppression of Tau spreading, linking axonal health to inflammatory mediators of the CNS. The authors further presented evidence that these microglia were able to spread Tau specifically through exosomes^[13]^, demonstrating how exosomes can spread neurite-damaging proteins between cells. Another group showed that primary mouse neurons produced exosomes that increased the proliferation and differentiation of neurons in mice *in vivo*, further highlighting the importance of exosomes in neuronal development^[104]^. These pieces of evidence show that information gained from *in vitro* studies of how exosomes modulate neurite integrity can extend to *in vivo* models.

Maintaining functional neurites is crucial to neuronal survival as well as to maintaining homeostasis. Though neurite formation and stability are affected by complex mechanisms, exosomal communication is a major mechanism by which these structures are modulated.

## CONCLUSION AND FUTURE DIRECTIONS

This review highlights the major progress made toward a better understanding of EV function in the CNS using *in vitro* models. While exosome research has seen a spectacular increase over the last decade, some challenges remain. More studies are needed to improve the knowledge of the different EV CNS subpopulations in terms of biogenesis and secretion, as well as uptake mechanisms, but also in terms of size, cargo composition, and specific markers. Since EVs play a significant dual role in cell-cell communication in neurodegenerative diseases, it is important to gain better knowledge about their potential beneficial and detrimental effects in different disease conditions. This could significantly contribute to the development of specific therapeutic avenues targeting, for example, the spread of inflammation and misfolded proteins that are characteristic of many neurodegenerative conditions.

There are many new research directions that can increase reproducibility in experimental systems as well as broaden our knowledge of EV biology. Co-cultures of neurons and glia, along with 3D organoid systems, can help elucidate how EV communication modulates different cell types in a more physiologically relevant model, potentially decreasing experiment-to-experiment variability. Utilizing organoids will be particularly interesting, as cells grown in 3D have been shown to have different rates of exosome secretion as well as different exosomal cargos^[105]^, and seem to produce EVs that are more closely related to EV produced in *in vivo* tissue systems than 2D cultures^[106]^. 3D organoids can also model different stages of organ development, which could potentially reveal differential EV characteristics in different stages of growth^[107,108]^.

Finally, there are many big questions in exosome research that remain unanswered. For example, does the endosomal origin of “true exosomes” cause them to have different effects when compared to small ectosomes or other non-endosomal extracellular vesicles?^[62]^ Could true endosomal exosomes have different effects on cell viability, inflammation, oxidative stress, and neurite health when compared to microvesicles? As a robust method has yet to be developed to ascertain the endosomal origin of EVs, this currently remains unknown. Moreover, in studying how exosomes modulate the inflammatory response of recipient cells, it will be important to determine whether exosomes carry cytokines in their cargo or whether the cytokines are attached to the outer surface of the membrane^[109]^.

## DECLARATIONS

### Copyright

© The Author(s) 2023.
